# Changing Epidemic of HIV and Syphilis Among Resident and Migrant Men Who Have Sex with Men in Jiangsu, China

**DOI:** 10.1038/s41598-017-08671-x

**Published:** 2017-08-25

**Authors:** Yuheng Chen, Weiming Tang, Lusi Chen, Lingen Shi, Xiaoyan Liu, Jinshui Xu, Haiyang Hu, Haitao Yang, Xiping Huan, Gengfeng Fu

**Affiliations:** 1 Institute for STI and HIV Control and Prevention, Jiangsu Provincial Center for Disease Control and Prevention, Nanjing, Jiangsu 210009 China; 2 University of North Carolina Project-China, Guangzhou, 510095 Guangdong China; 3 School of Public Health Southeast University, Nanjing, Jiangsu 210009 China; 4 Jiangsu Institute of Parasitic Diseases, Wuxi, Jiangsu 214064 China

## Abstract

Men who have sex with men (MSM) in China face high rates of HIV and syphilis infection exacerbated by internal migration. Studies on the differences of HIV and syphilis epidemics changing trends in high-risk behaviors and geographic distribution between resident and migrant MSM in Jiangsu, China were conducted. MSM were recruited from 14 surveillance sites in the serial cross-sectional study. Data on demographics, sexual behaviors, HIV and syphilis prevalence were collected. Participants were classified as residents or migrants based on household registration. During 2010–2014, 19,750 MSM were investigated. Engaged in anal sex (76.3% to 80.2%, P < 0.01) as well as received HIV-related services (72.1% to 79.2%, P < 0.01) were increasing. In contrast, engaged in commercial anal sex with males (7.4% to 5.0%, P < 0.01) and drug use (1.6% to 0.8%, P < 0.01) were decreasing. HIV prevalence ranged between 8.6% to 9.6%, while syphilis prevalence decreased over time (13.4–6.8%, P < 0.01). Further, we found that migrant MSM were more likely to engage in condomless anal sex, also had a higher HIV and syphilis prevalence than resident. During the study period, while syphilis prevalence decreased, higher rates of risk behaviors among migrant MSM called for targeted intervention strategies to reduce the HIV transmission.

## Introduction

Men who have sex with men (MSM) represent a high-risk group for the transmission of HIV/AIDS and syphilis, mainly due to low rates of condom use and high rates of multiple partners^1^. Over the past few years, MSM have become the highest-risk group for HIV infection in China^2^, with the rate of HIV infection rising annually. For example, the HIV/AIDS prevalence among MSM increased from 2.5% in 2006 to 7.8% in 2014^3^. The prevalence of syphilis among MSM also remains high, with a prevalence of 20.2% in Beijing^4^ and 11.4% in Zhejiang^5^. High-risk behaviors and lower coverage of preventive measures may be the potential driving force in the spread of HIV and sexually transmitted infections (STI)^6–8^.

It was estimated that there are more than 245 million internal migrants in China, accounting for 20% of the total population in 2013^1^. Migration in China seems to be temporary and unstable^9^. The majority of migrants do not have legal permanent urban residency status and receive less social welfare such as medical and educational benefits than permanent residents^10^. Previous studies have demonstrated that migration may be an important factor to the spread of HIV/STIs in China^11–13^. Migration functions as a “bridge” with lax social control, social isolation, and selective migration^14^, allowing the spread of HIV/STIs from high-risk groups to general population^15^. Furthermore, an epidemiologic study indicated that migration is more common among MSM, while migrant MSM are more likely to provide sexual services for money and engage in condomless sex, as compared with resident MSM^16, 17^. Because of the better employment opportunities, more comfortable living environment, and less discrimination^18^, large metropolitan and developed areas are more likely to attract MSM^19^. As one of the most densely populated and economically developed areas in eastern China, Jiangsu borders other less developed provinces and attracts a large number of MSM migrants^20^. The high rates of migration and sexual risk behaviors among MSM are challenges for HIV/STIs control in China. More control efforts should focus on migrant MSM, who are vulnerable to HIV/STIs.

This study aimed to evaluate the changing trends of HIV and syphilis epidemics, and risk behaviors among resident and migrant MSM in Jiangsu, China.

## Study Methods

### Sample size

Based on WHO recommendations and experience from other countries^21–23^, as well as the suggestion of Chinese CDC, a minimum sample size of 250 to 400 participants was required for each essential surveillance site. The sample size calculation has been reported elsewhere^18^. In short, the sample size was calculated using the following formula: N = [4*z_α_^2^ * P (1 − P)]/W^2^, wherein, z_α_ is a factor that corresponds to the desired confidence interval (for a 95% confidence level, z_α_ = 1.96); P is the expected proportion of patients with the outcome; W is the width of the interval, for example, the width for a margin of error of +/−3% is 0.06^22, 23^.

### Recruitment and Ethical Approval

Data collections were conducted between April and August of each year from 2010 through 2014. To be eligible, participants needed to be at least 18 years old, born biologically male and report having oral or anal sex with men in the previous year. MSM who had been diagnosed HIV positive in the past should also be incorporated into the survey. Convenience sampling methods (snowball sampling, venue-based sampling, and online recruitment) were used to recruit participants. Investigators within the MSM community found potential subjects in MSM activity venues, including commercial entertainment locations (bars and clubs) and public places (parks and public restroom) and invited them to participate in the study. Recruitment information was also placed on Internet forums, online chat rooms, and instant messaging software used by MSM. To ensure the quality of the survey, in each of the study site, the same sampling design and study protocol, which include comprehensive quality control plan, were followed and staff were trained as per the same training module.

The survey was approved by the ethics committees of the Jiangsu Provincial Center for Disease Control and Prevention (JSCDC). A signed informed consent was obtained from each participant for the questionnaire survey and blood collection. Each participant was reminded of the right to decline participation or quit the study at any time. All methods were performed in accordance with the relevant guidelines and regulations that were approved by the Ethical Committee of Jiangsu CDC.

### Demographic and Behavioral Measures

We collected information regarding demographics, sexual risk behaviors, drug use (refers to traditional drugs) and the receiving HIV-related services from the participants by using a structured, self-administered questionnaire. According to the self-reported official household registration, participants were classified as residents or non-residents (migrants). If participants reported that they were from a different province or foreign country, they were defined as migrants. Otherwise, participants were considered as residents, consistent with similar past studies^24–26^. Demographic questions included age, marital status, education level, the length of stay in the local area, and common venues for finding partners.

Participants were asked to recall their sexual activities and behaviors in the last six months, including non-commercial sex with males, commercial anal sex with a male, vaginal sex with a female, and use of a condom during these sexual activities. Other behaviors, including injection and non-injection drug use, were also included in the questionnaire. Participants were also asked about their exposure to HIV/AIDS preventive services, including free condom services, HIV/AIDS counseling and detection, methadone maintenance therapy, clean needle exchange and peer education. Participants who had accepted one of these services in the last year were categorized as having received HIV/AIDS related services.

### Biological testing for HIV and Syphilis

Five ml. of venous blood (anticoagulated) was collected from each participant for HIV and syphilis antibody testing, using the standard protocol and laboratory methods^27^. HIV antibodies were screened using a rapid test (Acon Biotec Co., Ltd), with positive screening tests confirmed using Western Blot (WB, HIVBLOT 2.2, Genelabs Diagnostics, Singapore). Syphilis antibodies were screened using the ELISA (Wantai Biopharmacy Co., Ltd) test and confirmed with TRUST (Wantai Biopharmacy Co., Ltd)^28^.

### Statistical analysis

All data were entered into the HIV/AIDS Sentinel Surveillance Network server using client software. After data had been exported and cleaned, SAS version 9.2^29^ and SPSS 23.0 were used for statistical analyses. Descriptive analysis was conducted to compare demographic characteristics, sexual behaviors, drug use, and use of HIV-related services, as well as to compare HIV and syphilis infection rates between resident and migrant MSM. The Cochran-Armitage trend test was used to examine the trend associated with these risk factors during the study period. Univariable and multivariable logistic regression models were conducted to compare the HIV/syphilis prevalence and related sexual risk behaviors, by comparing the data collected in 2014 to 2010. In univariate analysis, each infection (HIV/syphilis) and various risk behaviors were defined as dependent variables, while time was treated as an independent variable. Multivariate logistic regression adjusted for age, marital status and education. Crude odd ratios and adjusted odd ratios (AOR), as well as their 95% confidence intervals (95% CI), were calculated to demonstrate the association between time and factors. Chi-square statistics were applied to test the differences between factors associated with sexual risk behaviors for HIV positive and negative individuals in each surveyed year. Epi Info software was used to determine and demonstrate the geographical distribution of the epidemics. Electronic maps were obtained from Jiangsu CDC.

## Results

### Demographic Characteristics

From 2010 to 2014, a total of 19,750 MSM participants were surveyed from 14 surveillance points in Jiangsu, ranging from 3,061 to 4,766 per year. Among the surveyed individuals, approximately 80.0% were residents, and 20.0% were migrants. The proportion of residents increased from 75.6% in 2010 to 82.7% in 2012 and then decreased to 80.5% in 2014.

The average age of the participant was 30.9 ± 10.4 years old. For participants under the age of 30, the proportion of migrants (64.5%) was higher than residents (53.6%). Approximately 58.8% of residents and 69.7% of migrants reported never having been married. About three-quarters of all participants had at least attained senior high school, with 79.4% among residents and 71.5% among migrants, respectively. Among both residents and migrants, the most common location for meeting partners was the Internet followed by bars and clubs. Residents were somewhat more likely than migrants to meet partners on the Internet, while migrants were more likely than residents to meet partners at bath centers (Table 1).

**Table 1:** Demographic Characteristics of MSM Recruited Between 2010 and 2014 in Jiangsu (N = 19,750).

Characteristics	2010		2011		2012		2013		2014		Total	
	(N = 3061)		(N = 3337)		(N = 4380)		(N = 4208)		(N = 4766)		(N = 19750)	
	Resident %	Migrants%	Resident %	Migrants%	Resident %	Migrants%	Resident %	Migrants %	Resident %	Migrants%	Resident %	Migrant%
**Percentage of the class**	75.6	24.4	79	21	82.7	17.3	80.7	19.3	80.5	19.5	80	20
**Age (years)**												
<*20*	5	4.8	6.8	4.6	5.1	4.9	6.1	5.9	4.6	8.1	5.4	5.8
*20–29*	45.1	56.9	50.4	63.6	48.5	56	48.1	61.2	48.3	56.6	48.2	58.7
*30–39*	27.5	25.6	23.3	22.3	25.8	27.1	25.9	23.2	23.5	21	25.1	23.7
*40–49*	16.7	9.5	14	7.4	14.3	9.6	13.2	6.9	15.3	9.5	14.6	8.6
≥50	5.7	3.2	5.5	2.1	6.5	2.4	6.7	2.7	8.3	4.8	6.7	3.1
**Marital status**												
*Single*	58.6	64.8	63.3	71.4	56.8	66.6	60.6	73	56.1	72.3	58.8	69.7
*Married*	33.9	28	32	26	38.8	31.2	33.2	22	38.3	22.7	35.6	25.8
*Divorced/ Widowed*	7.5	7.3	4.7	2.6	4.4	2.2	6.2	5.1	5.6	5.1	5.6	4.5
**Highest level of education**												
*Junior high school or lower*	22.8	34.5	19.4	22.6	21.9	28.2	21.5	30.5	18.1	26.8	20.6	28.5
*Senior high school or above*	77.2	65.5	80.6	77.4	78.1	71.8	78.5	69.5	81.9	73.2	79.4	71.5
**Length of time living in current location**												
*Less than one year*	19.9	47.3	18.5	39.1	12.5	35.9	11.2	37.8	8.8	36	13.4	39
*One year or more*	80.1	52.7	81.5	60.9	87.5	64.1	88.8	62.2	91.2	64	86.6	61
**Partner seeking Venues**												
*Internet*	24.5	21.4	34.5	36.7	47.6	40.7	40.2	24.3	62.5	29.3	44	30.3
*Bar/Club*	35.4	26.2	23.9	18.6	29.2	27.8	36.6	39.3	18.6	31.3	28.2	29
*Bath center*	24.7	36.9	24	28.4	16.5	26.4	7.3	11.9	6.7	16.1	14.6	23.3
*Public places*	0.4	0.1	2.2	0.6	0.6	0.5	2.8	2.7	0.2	0.2	1.2	0.8
*Other*	15	15.3	15.3	15.7	6.1	4.6	13.1	21.8	11.9	23.2	11.9	16.5

### Sexual Risk Behaviors

About four-fifths of the participants had engaged in anal sex in the last six months, with an increasing trend among both residents and migrants during the study period (P < 0.01, AOR = 1.24, 95% CI:1.09–1.40 in residents; AOR = 1.47, 95% CI:1.15–1.87 in-migrants). Increases were observed among resident MSM in rates of condom use during most recent anal sex with a male partner (P < 0.01, AOR = 1.29, 95% CI:1.13–1.46) and consistent condom use during anal sex in the last six months (P < 0.01, AOR = 1.19, 95% CI:1.05–1.34), while no statistically significant changes in these two behaviors were observed among migrants. However, the proportion reporting these three behaviors in migrants was higher than in residents for the majority of the surveyed period.

The trend in commercial anal sex for both residents and migrants decreased during the study period (P < 0.01), with a somewhat higher proportion of migrants (7.2%) participating, as compared to residents (4.4%).

About 29.3% (5,777/19,700) participants indicated that they had engaged in vaginal intercourse with a female at least once in the last six months. The proportion of participants reporting vaginal sex decreased among migrants (P < 0.01, AOR = 0.61, 95% CI: 0.47–0.78). The proportions of residents who used a condom during their most recent vaginal intercourse increased from 42.0% in 2010 to 50.2% in 2014 (P < 0.01, AOR = 1.48, 95% CI: 1.22–1.80).

A significant decreasing trend of drug use was observed in both residents (AOR = 0.57, 95% CI: 0.34–0.96) and migrants (AOR = 0.39, 95% CI: 0.17–0.88). While the proportion who received any HIV-related services increased consistently over time in both residents (AOR = 1.45, 95% CI:1.29–1.64) and migrants (AOR = 1.48, 95% CI:1.18–1.85) (Table 2).

**Table 2:** Behaviors, Receipt of Services and Serological Detection of MSM Changing across time during 2010 and 2014 in Jiangsu (N = 19,750).

Variables	2010	2011	2012	2013	2014	Overall	*P* for Trend	Crude OR^#^ (95% CI)		Adjusted OR* (95% CI)		*P* for Adjusted Model
	(%)	(%)	(%)	(%)	(%)	(%)						
**Engaged in anal sex in the last six months**												
Residents	76.2	78.2	73.8	77.8	79.6	77.2	<0.01	1.22	(1.08–1.38)	1.24	(1.09–1.40)	<0.01
Migrants	76.5	83.6	84.7	83.6	82.4	82.2	<0.01	1.44	(1.13–1.83)	1.47	(1.15–1.87)	<0.01
All	76.3	79.4	75.7	78.9	80.2	78.2	<0.01					
**Used condom during last anal intercourse with male**												
Residents	65.0	72.9	72.4	72.2	70.4	70.9	<0.01	1.28	(1.13–1.45)	1.29	(1.13–1.46)	<0.01
Migrants	68.3	71.4	72.8	67.6	69.9	69.9	0.43	1.08	(0.85–1.36)	1.08	(0.86–1.37)	0.51
All	65.8	72.6	72.5	71.2	70.3	70.7	0.02					
**Consistent condom use during anal sex with male in the last six months**												
Residents	36.7	44.0	41.9	43.0	40.9	41.5	0.06	1.19	(1.06–1.35)	1.19	(1.05–1.34)	<0.01
Migrants	43.3	45.9	47.3	40.1	41.7	43.5	0.06	0.93	(0.75–1.16)	0.94	(0.75–1.17)	0.55
All	38.3	44.5	43.0	42.4	41.0	41.9	0.29					
**Engaged in commercial anal sex with male in the last six months**												
Residents	6.3	5.2	3.8	3.0	4.5	4.4	<0.01	0.70	(0.54–0.90)	0.75	(0.58–0.98)	0.03
Migrants	10.8	9.0	5.9	3.5	7.3	7.2	<0.01	0.66	(0.45–0.96)	0.69	(0.47–1.02)	0.07
All	7.4	6.1	4.2	3.1	5.0	5.0	<0.01					
**Used condom during last commercial anal intercourse with male**												
Residents	75.2	83.0	68.7	79.2	85.8	78.9	0.05	1.99	(1.03–3.84)	1.98	(1.01–3.88)	0.05
Migrants	86.7	90.4	67.6	79.2	82.1	82.5	0.12	0.71	(0.26–1.95)	0.70	(0.24–2.00)	0.50
All	79.4	85.4	68.4	79.2	84.7	80.0	0.26					
**Consistent condom use during commercial anal sex in the last six months with male**												
Residents	47.7	60.7	42.7	62.8	49.3	52.1	0.48	1.07	(0.64–1.77)	1.08	(0.64–1.81)	0.79
Migrants	63.3	67.3	47.4	41.7	58.9	58.3	0.11	0.83	(0.39–1.76)	0.67	(0.30–1.47)	0.32
All	53.3	62.9	44.0	57.8	52.1	54.0	0.23					
**Engaged in vaginal sex in the last six months**												
Residents	30.2	28.6	33.5	28.4	29.7	30.2	0.23	0.98	(0.87–1.09)	0.89	(0.79–1.02)	0.08
Migrants	30.7	28.3	29.6	22.3	20.0	25.8	<0.01	0.56	(0.45–0.71)	0.61	(0.47–0.78)	<0.01
All	30.3	28.6	32.8	27.2	27.8	29.3	<0.01					
**Used condom during last vaginal intercourse**												
Residents	42.0	49.3	59.0	55.6	50.2	52.2	<0.01	1.39	(1.15–1.69)	1.48	(1.22–1.80)	<0.01
Migrants	44.2	46.9	61.0	58.1	48.6	51.7	0.03	1.19	(0.81–1.76)	1.26	(0.84–1.89)	0.27
All	42.5	48.8	59.3	56.0	50.0	52.1	<0.01					
**Consistent condom use during vaginal sex in the last six months**												
Residents	24.5	28.3	21.8	25.6	24.7	24.7	0.36	1.01	(0.81–1.26)	1.05	(0.84–1.32)	0.68
Migrants	27.6	30.6	29.1	36.9	30.3	30.7	0.13	1.14	(0.74–1.75)	1.15	(0.73–1.80)	0.55
All	25.3	28.8	23.0	27.3	25.5	25.8	0.43					
**Used drug in lifetime**												
Residents	1.3	1.3	0.9	0.7	0.7	0.9	<0.01	0.52	(0.31–0.88)	0.57	(0.34–0.96)	0.04
Migrants	2.4	1.0	1.2	0.9	1.0	1.3	<0.01	0.40	(0.18–0.89)	0.39	(0.17–0.88)	0.02
All	1.6	1.3	0.9	0.7	0.8	1.0	<0.01					
**Received any HIV-related services in last year**												
Residents	72.5	78.2	76.7	81.7	79.4	78.1	<0.01	1.47	(1.30–1.66)	1.45	(1.29–1.64)	<0.01
Migrants	71.1	75.3	73.4	82.1	78.4	76.3	<0.01	1.48	(1.19–1.85)	1.48	(1.18–1.85)	<0.01
All	72.1	77.6	76.1	81.8	79.2	77.7	<0.01					
**HIV prevalence**												
Residents	8.1	7.1	9.0	7.3	8.8	8.1	0.13	1.10	(0.91–1.32)	1.12	(0.93–1.36)	0.23
Migrants	11.1	10.9	12.1	14.1	9.2	11.4	0.34	0.81	(0.59–1.11)	0.83	(0.60–1.14)	0.25
All	8.9	7.9	9.6	8.6	8.9	8.8	0.30					
**Syphilis infection rate**												
Residents	12.8	8.9	6.5	5.5	6.5	7.6	<0.01	0.48	(0.40–0.57)	0.48	(0.40–0.57)	<0.01
Migrants	15.4	9.4	7.9	5.8	8.1	9.2	<0.01	0.48	(0.35–0.66)	0.48	(0.35–0.65)	<0.01
All	13.4	9.0	6.8	5.5	6.8	7.9	<0.01					

Note: ^#^Odds Ratio was calculated by direct comparing the year 2014 to the year 2010 (reference group); *Multivariate models were adjusted for the following variables: age, marital status (ref = married), education. P < 0.01.

### HIV/Syphilis Prevalence and Trend

1738 participants tested positive for HIV, with an overall HIV prevalence of 8.8%. There was no obvious change in HIV prevalence among the two groups during the study period. However, it is important to note that the HIV infection rate was higher among migrants (11.4%), as compared to residents (8.1%).

**Figure 1:**
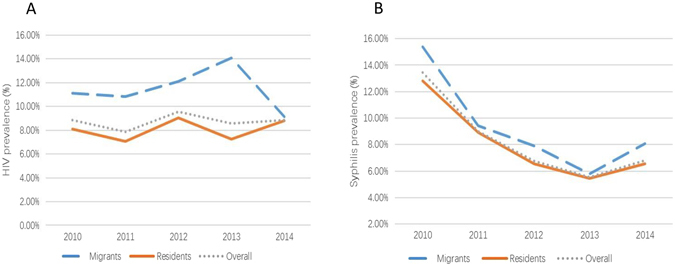
Trend of HIV (**A**) and syphilis (**B**) prevalence (overall, migrants, residents) for the participants during 2010 to 2014 in Jiangsu. (Created by Microsoft Excel, Redmond, Washington, the United States available at https://www.microsoftstore.com.cn/c/office?Icid=StoreNavi_Office).

While HIV prevalence was stable, the prevalence of syphilis decreased significantly (from 12.8% in 2010 to 6.5% in 2014, P < 0.01) among both resident MSM (P < 0.01, AOR = 0.48; 95% CI: 0.40–0.57) and migrant MSM (P < 0.01, AOR = 0.48; 95% CI: 0.35–0.65). (Table 2) Syphilis prevalence among migrants was also consistently higher than residents during the study period (Fig. 1).

### Behaviors correlated with HIV status

Compared to HIV-negative participants, the majority of MSM in the HIV-positive group had more anal sex in last six months (Table 3). From 2011 to 2014, a higher proportion of HIV-negative MSM used a condom during their last anal sex, and similar results were also observed for consistent condom use during the last six months. In addition, a significantly smaller proportion of HIV-positive participants engaged in commercial anal sex during the study period. Vaginal sex in the last six months was associated with HIV infections in the 2010 and 2012 surveys, while consistent condom use during vaginal sex in the last six months was associated with HIV infection in 2014. We also found that there was a link between lifetime drug use and HIV infection among HIV-positive MSM in 2011 and 2014 surveys (Table 3).

**Table 3:** HIV-Related Services and Behaviors for HIV negative and positive MSM* in Jiangsu (2010–2014, N = 19,750).

Variable	2010 (%)		2011 (%)		2012 (%)		2013 (%)		2014 (%)		Overall	
**Engaged in anal sex in the last six months**												
HIV−	76.2	(2121/2783)	78.8	(2408/3057)^a^	75.0	(2960/3948)^a^	78.2	(3007/3846) ^a^	80.1	(3485/4351)	77.7	(13981/17985)^a^
HIV+	76.4	(207/271)	86.2	(225/261)	82.3	(345/419)	87.0	(314/361)	81.1	(344/424)	82.7	(1435/1736)
**Used condom during last anal intercourse with male**												
HIV−	66.1	(1380/2088)	73.2	(1739/2377)^a^	73.1	(2123/2904)^a^	72.4	(2173/3001)^a^	71.3	(2475/3473)^a^	71.4	(9890/13843)^a^
HIV+	63.4	(128/202)	66.5	(149/224)	67.3	(231/343)	60.1	(188/313)	61.0	(210/344)	63.5	(906/1426)
**Consistently used condom during anal sex in the last six months**												
HIV−	38.6	(801/2073)	45.6	(1084/2376) ^a^	43.8	(1267/2891)^a^	44.1	(1323/3002)^a^	42.2	(1463/3463)^a^	43.0	(5938/13805)^a^
HIV+	34.8	(69/198)	32.0	(71/222)	35.8	(123/344)	26.6	(83/312)	28.2	(97/344)	31.2	(443/1420)
**Engaged in commercial anal sex with male in the last six months**												
HIV−	7.2	(150/2116)	6.3	(151/2398)	4.1	(122/2965)	3.1	(92/3007)	4.7	(165/3474)^a^	4.9	(683/13960)
HIV+	8.8	(18/204)	3.6	(8/224)	5.3	(18/342)	3.2	(10/314)	7.8	(27/344)	5.7	(81/1428)
**Used condom during last commercial anal intercourse**												
HIV−	79.7	(118/148)	85.4	(129/151)	67.8	(82/121)	80.2	(73/91)	89.0	(145/163)^a^	81.2	(547/674)^a^
HIV+	76.5	(13/17)	85.7	(6/7)	73.3	(11/15)	70.0	(7/10)	59.3	(16/27)	69.7	(53/76)
**Consistently used condom during commercial anal sex in the last six months**												
HIV−	51.0	(76/146)^a^	62.3	(94/151)	43.1	(50/116)	58.7	(54/92)	55.8	(91/163)^a^	54.4	(365/671)
HIV+	72.2	(13/18)	75.0	(6/8)	50.0	(9/18)	50.0	(5/10)	29.6	(8/27)	50.6	(41/81)
**Engaged in vaginal sex in the last six months**												
HIV−	29.6	(823/2777)^a^	28.4	(869/3056)	33.5	(1324/3951)^a^	27.5	(1057/3843)	28.2	(1226/4343)	29.5	(5299/17970)
HIV+	37.4	(101/270)	30.3	(79/261)	26.1	(109/417)	24.2	(87/360)	24.2	(102/422)	27.6	(478/1730)
**Used condom during last vaginal intercourse**												
HIV−	42.5	(348/819)	48.9	(417/853)	59.9	(777/1297)	56.2	(591/1052)	49.6	(606/1221)	52.3	(2739/5242)
HIV+	42.4	(42/99)	48.1	(38/79)	53.2	(58/109)	54.0	(47/87)	55.9	(57/102)	50.8	(242/476)
**Consistently used condom during vaginal sex in the last six months**												
HIV−	24.8	(200/808)	29.1	(247/849)	22.8	(294/1292)	26.7	(281/1054)	24.7	(301/1218)^a^	25.3	(1323/5221) ^a^
HIV+	28	(28/94)	25.3	(20/79)	26.2	(28/107)	35.6	(31/87)	34.7	(35/101)	30.3	(142/468)
**Used drug in lifetime**												
HIV−	1.7	(46/2782)	1.2	(35/3040)^a^	0.9	(37/3949)	0.7	(26/3845)	0.6	(26/4334)^a^	0.9	(170/17950)^a^
HIV+	1.1	(3/270)	2.7	(7/261)	0.7	(3/415)	1.1	(4/361)	2.4	(10/421)	1.6	(27/1728)
**Received any HIV-related services in last year**												
HIV−	71.5	(1997/2792)	77.7	(2391/3076)	76.4	(3028/3965)	82.3	(3166/3846)^a^	80.1	(3485/4351)^a^	78.0	(14067/18030)^a^
HIV+	78.7	(214/272)	76.0	(199/262)	73.7	(309/419)	76.5	(276/361)	71.0	(301/424)	74.7	(1299/1738)

Note: *Those information were collected before HIV diagnosis. ^a^Represents statistical significance, P < 0.05.

### Geographic distributions

**Figure 2:**
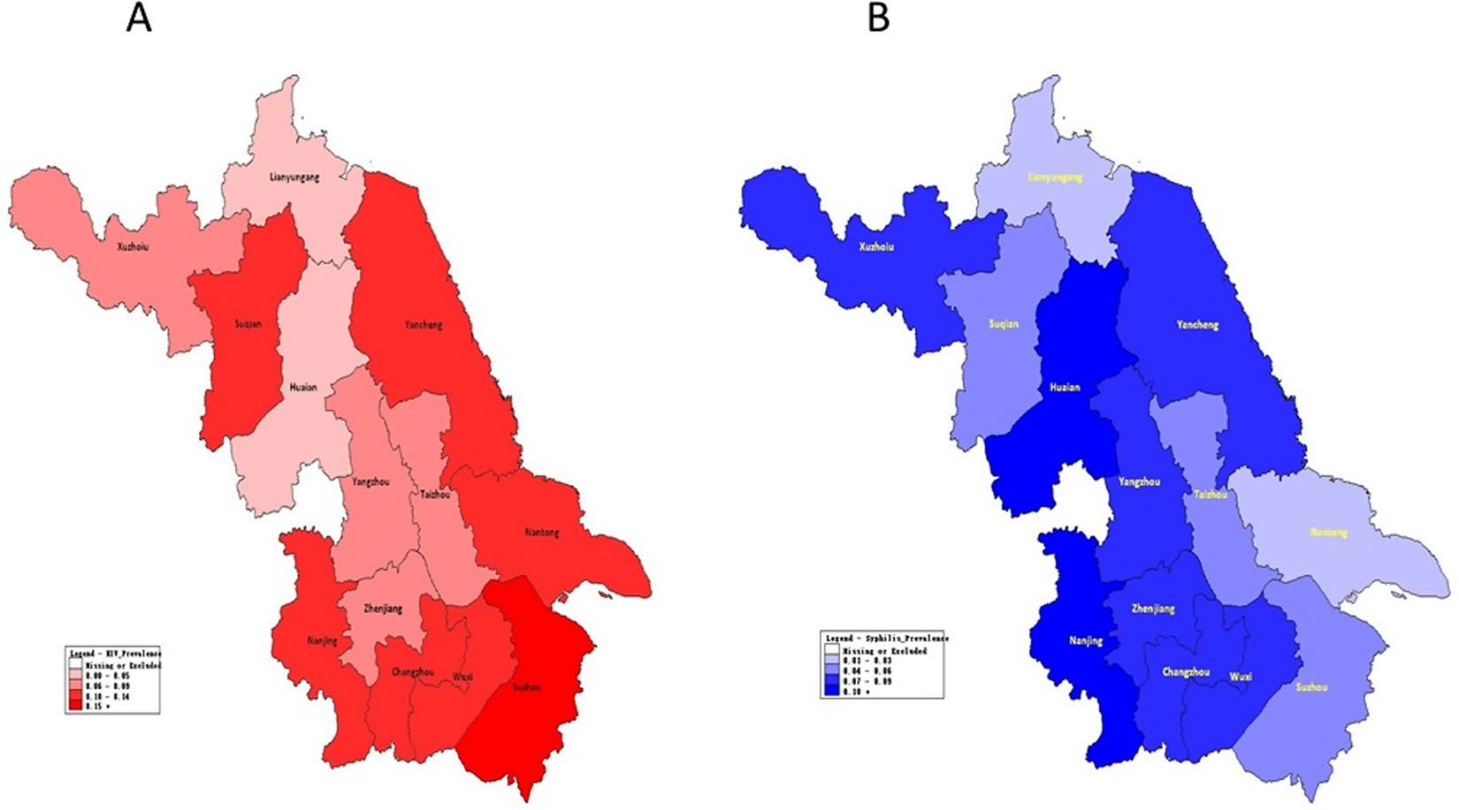
Map of Jiangsu showing the geographical distribution of HIV (**A**) and syphilis (**B**) prevalence among MSM in 2014 in 13 cities. **(**Created by Epi Info 3.5.1 software, Centers for Disease Control and Prevention, Atlanta, GA, USA, available at http://www.cdc.gov/epiinfo/index.html **)**.

Geographic distribution of HIV, syphilis seropositivity in 13 sampling cities during 2014 was presented in Fig. 2, color-coded to represent the geographical differences in HIV and syphilis prevalence. Overall, Suzhou had the highest HIV prevalence (>16%) in Jiangsu province, and seven cities had HIV infection rates over 10%. For syphilis, Nanjing and Huai’an reported prevalence over 10%. Compared to Southern Jiangsu, migrants in Central Jiangsu and residents in Northern Jiangsu has lower HIV prevalence, while people in Central Jiangsu had the lowest syphilis prevalence (Table 4).

**Table 4:** Odds ratios Between Human Immunodeficiency Virus (HIV) and Syphilis Among MSM in different regions of Jiangsu, 2014 (n = 4,766).

Variable	Region^a^	Migrants		Residents		Overall	
		Crude OR^#^ (95% CI)	Adjusted OR^#^ (95% CI)	Crude OR^#^ (95% CI)	Adjusted OR^#^ (95% CI)	Crude OR^#^ (95% CI)	Adjusted OR^#^ (95% CI)
HIV	**South Jiangsu**	Ref					
	**Central Jiangsu**	0.37 (0.18–0.75)^*^	0.3 (0.14–0.64)^*^	0.88 (0.67–1.17)	0.87 (0.65–1.16)	0.77 (0.59–0.99)	0.74 (0.57–0.96)
	**North Jiangsu**	0.51 (0.20–1.29)	0.52 (0.20–1.33)	0.63 (0.49-0.82)^*^	0.57 (0.43–0.74)^*^	0.61(0.48–0.78)^*^	0.58 (0.45–0.74)^*^
Syphilis	**South Jiangsu**	Ref					
	**Central Jiangsu**	0.71 (0.38–1.32)	0.59 (0.31–1.13)	0.65 (0.46–0.93)	0.61 (0.43–0.88)*	0.66 (0.48–0.89)*	0.6 (0.44–0.81)^*^
	**North Jiangsu**	0.8 (0.33–1.92)	0.82 (0.34–1.97)	0.77 (0.58–1.02)	0.7 (0.52–0.94)	0.74 (0.57–0.96)	0.68 (0.52–0.88)^*^

Note: ^a^South Jiangsu: Nanjing, Wuxi, Changzhou, Suzhou, Zhenjiang; Central Jiangsu: Nantong, Yangzhou, Taizhou; North Jiangsu: Xuzhou, Lianyungang, Huai’an, Yancheng, Suqian.

^#^Odds Ratio was calculated by direct comparing region Central Jiangsu and North Jiangsu to South Jiangsu (reference group); Multivariate models were adjusted for the following variables: age, marital status (ref = married), education. *Represents statistical significance. P < 0.01.

## Discussion

Our study found a higher prevalence of HIV and syphilis and higher rates of some high-risk behaviors in migrant MSM than in resident MSM. Knowledge of trends of HIV/STI prevalence and related sexual behaviors in migrant and resident MSM is critical for HIV/STI control efforts. In China, many HIV testing and care programs are targeted toward the general MSM population^4, 30, 31^, but few focus specifically on migrant MSM. This study extends the literature by exploring the trend of HIV and syphilis prevalence, related sexual behaviors, and geological distribution among MSM in Jiangsu. From the study, we found a decreasing trend of syphilis prevalence, while HIV prevalence was consistent.

In China, migrant population is often seen as poor, poorly educated, rural farmers or laborers who seek for a better job in large cities^32^. In our study, one-fifth of MSM participants is migrants. When they are living in a new city without sufficient HIV knowledge, family discipline and social support, they are more prone to make a sexual decision without constraint^12, 25, 33^, and more likely to engage in high-risk sexual behaviors and acquire HIV/STIs^34–36^. The results indicated that only resident MSM had an increasing trend in condom use during last anal sex and inconsistent condom use in last six months, while migrant MSM did not show a consistent trend across time. Our study also found that compared with resident MSM, migrant MSM had higher HIV and syphilis prevalence, more frequent anal sexual behaviors, and higher lifetime drug use rates, whichever it is, consistent with a previous study of MSM in China^37^. Further studies on evaluating which factors had contributed to the high HIV and syphilis infections among migrants are needed.

The overall HIV prevalence from our study was about 8.8%, which was higher than a previous survey in Jiangsu^38^ and higher than findings from two meta-analyses (6.5% in 2013^39^ and 7.7% in 2013^3^, respectively). HIV prevalence among surveyed MSM in Jiangsu was higher than that of a cross-sectional survey^32^ and five years of accompanying analysis on HIV/AIDS in China^2^. Unlike findings from other studies^4, 18, 40^, the trend of HIV prevalence in our study did not change during the study period but remained at a high level. This high prevalence is particularly concerning given our study spanned five years and included a large sample of MSM.

Syphilis prevalence among MSM in our study was about 7.9%, which was much lower than several previous studies of Jiangsu^28, 38, 41^ and also lower than other previous surveys of China^32, 42^. Unlike the trend of HIV prevalence, syphilis prevalence decreased consistently, which is consistent with the previous literature^18^. One potential explanation for this phenomenon is the expansion of syphilis testing and control programs as well as the improving of other healthcare service^28, 40^, while syphilis is a curable disease but not HIV. Syphilis prevention strategies included early screening and appropriate treatments^6^. Among the high-risk population, the coverage of screening and treatment for syphilis were increased in recent years in China^4^. Several MSM-targeted interventions were implemented in parallels, such as 100% condom promotion, behavioral intervention, and expansion of syphilis testing and treatment^7^. These measures may have led to a decline in syphilis prevalence among MSM. These prevention strategies may also have contributed to HIV control, which may explain why HIV epidemic was stable during the study period. As we know, Syphilis infection is at greater risk of HIV infection^43^, the high rate of Syphilis may suggest an increasing rate of HIV among MSM. However, the geographic distribution of HIV and Syphilis are not consistent in Jiangsu province, while the specific reasons for this inconsistent are still not clear, curable of syphilis could be one potential reason^44^.

Our study found that HIV-positive individuals are more likely to engage in anal sex, but less likely to use condoms consistently, as compared to HIV-negative participants. Previous studies demonstrated that condomless sexual activity is a risk factor for HIV infections in MSM^45, 46^, particularly these participants who knows they were HIV-positive when a condom was not used during sexual intercourse, the virus will be transmitted easily through the anal intercourse^47^. Our study also indicated that even migrant MSM and resident MSM have comparable behaviors on condom use, migrant MSM tend to have a high prevalence of HIV. One potential reason for this phenomenon is migrants tend to have more sexual partners and tend to have a set of sexual partners with greater STI risk behaviors^26^, such as more secondary sexual partners (partners other than the index) and more receptive anal intercourse.

Due to the unregistered residential status, migrant MSM tends to have less opportunity to access many healthcare services and are not well covered by many HIV/STI programs in most places in China^48, 49^. For example, previous studies show that migrant MSM faces barriers to accessing adequate health services^9, 50, 51^. In addition, the healthcare system in China has lower rates of health services coverage for migrants as compared to residents, especially for HIV prevention services^52^. Meanwhile, the migrants are also rarely utilizing these medical resources^52^. Therefore, more efforts are needed to promote interventions targeting migrant MSM. First, the public sector should increase the social supports and help to build and strengthen healthcare system to cover the whole population of China. Second, specific attention is required tailored behavioral interventions program on migrant MSM, like condom promotion, peer education. Third, a free and easily accessible HIV screening is necessary to be provided for migrant MSM as well as the treatment for HIV/STIs.

Our study has several limitations. First, because of the study design, lack of temporality prevented us from studying the relationship of demographic and high-risk factors for HIV/STI epidemics between residents and migrants. Second, MSM living with HIV with knowing status may be no longer willing to participate in sentinel surveillance, and the HIV prevalence reported in our study tends to be understated. Third, since most of our data were self-reported, social desirability bias can occur. Fourth, when participants recalled behavior over the last six months, misclassification of exposures may also occur. Last but not least, we did not consistently collect data on whether participants knew their HIV and syphilis status, which limited our ability to adjust this in our data analysis.

## Conclusion

This study suggests that prevalence of HIV among MSM in Jiangsu remains high, and HIV control remains an ongoing challenge in the future. In addition, this study demonstrates that migrant MSM compared to resident MSM were more likely to have high sexual risk behaviors. To decrease the HIV/STIs burden among MSM in Jiangsu, especially among migrant MSM, targeted intervention programs should be developed, and comprehensive intervention strategies should be enhanced to ensure that the needs of this high-risk population are met.
